# A pre-post study of a multi-country scale up of resuscitation training of facility birth attendants: does Helping Babies Breathe training save lives?

**DOI:** 10.1186/s12884-016-0997-6

**Published:** 2016-08-15

**Authors:** Roopa M. Bellad, Akash Bang, Waldemar A. Carlo, Elizabeth M. McClure, Sreelatha Meleth, Norman Goco, Shivaprasad S. Goudar, Richard J. Derman, Patricia L. Hibberd, Archana Patel, Fabian Esamai, Sherri Bucher, Peter Gisore, Linda L. Wright, for the HBB Study Group, Roopa M. Bellad, Shivaprasad S. Goudar, Bhala S. Kodkany, Manjunath S. Somannavar, Veena R. Herekar, Sunil S. Vernekar, Narayan V. Honnungar, Chandrashekhar C. Karadiguddi, Akash Bang, Archana Patel, Savita R. Bhargava, Kunal Kurhe, Rakesh Kukde, Peter Gisore, Fabian Esamai, David Muyodi, Coletta Makokha, Waldemar A. Carlo, Elizabeth M. McClure, Sreelatha Meleth, Dennis D. Wallace, Norman Goco, Janet L. Moore, Patricia L. Hibberd, Edward A. Liechty, Sherri Bucher, Richard J. Derman, Frances J. Jaeger, Linda L. Wright

**Affiliations:** 1 KLE University’s Jawaharlal Nehru Medical College, Belgaum, Karnataka India; 2 Mahatma Gandhi Institute of Medical Sciences, Sewagram, Maharashtra India; 3 University of Alabama at Birmingham, Birmingham, AL USA; 4 RTI International, Durham, NC USA; 5 Department of Obstetrics and Gynecology, Christiana Care Health Services, Newark, DE USA; 6 Massachusetts General Hospital, Boston, MA USA; 7 Lata Medical Research Foundation, Nagpur, India; 8 Indira Gandhi Government Medical College, Nagpur, India; 9 Department of Child Health and Paediatrics, Moi University School of Medicine, Eldoret, Kenya; 10 School of Medicine, Indiana University, Indianapolis, IN USA; 11 Eunice Kennedy Shriver National Institute of Child Health and Human Development, Bethesda, MD USA; 12 5800 Nicholson Lane, #1206, Rockville, MD 20852 USA

## Abstract

**Background:**

Whether facility-based implementation of Helping Babies Breathe (HBB) reduces neonatal mortality at a population level in low and middle income countries (LMIC) has not been studied. Therefore, we evaluated HBB implementation in this context where our study team has ongoing prospective outcome data on all pregnancies regardless of place of delivery.

**Methods:**

We compared outcomes of birth cohorts in three sites in India and Kenya pre-post implementation of a facility-based intervention, using a prospective, population-based registry in 52 geographic clusters. Our hypothesis was that HBB implementation would result in a 20 % decrease in the perinatal mortality rate (PMR) among births ≥1500 g.

**Results:**

We enrolled 70,704 births during two 12-month study periods. Births within each site did not differ pre-post intervention, except for an increased proportion of <2500 g newborns and deliveries by caesarean section in the post period. There were no significant differences in PMR among all registry births; however, a post-hoc analysis stratified by birthweight documented improvement in <2500 g mortality in Belgaum in both registry and in HBB-trained facility births. No improvement in <2500 g mortality measures was noted in Nagpur or Kenya and there was no improvement in normal birth weight survival.

**Conclusions:**

Rapid scale up of HBB training of facility birth attendants in three diverse sites in India and Kenya was not associated with consistent improvements in mortality among all neonates ≥1500 g; however, differential improvements in <2500 g survival in Belgaum suggest the need for careful implementation of HBB training with attention to the target population, data collection, and ongoing quality monitoring activities.

**Trial registration:**

The study was registered at ClinicalTrials.gov: NCT01681017.

## Background

Helping Babies Breathe, a modification of the Neonatal Resuscitation Program specifically designed for low resource settings, focuses on establishing breathing during the “Golden Minute” after birth by immediately drying/stimulating the baby to initiate spontaneous respiration, followed by bag and mask ventilation, as needed [1]. It has been shown to be effective in several low and middle income countries LMIC in reducing early neonatal deaths for infants born in health facilities [2, 3]. However, in many such countries, a large percentage of infants are born outside of health facilities. It is not known what effect an HBB program would have on overall neonatal mortality of the population in diverse settings, including those with large numbers of home births.

A major emphasis of the World Health Organization has been to encourage women to choose to deliver in a health facility with a skilled birth attendant (BA) [4]. The reasons women choose to deliver at home rather than in hospitals are multifactorial and complex; however, a recurring theme is mistrust of the health care system and lack of confidence that a facility birth will result in a better outcome than a home birth [5–7].

It is unknown whether facility based interventions, such as HBB, will reduce neonatal mortality at a population level in LMIC with large numbers of home births. We therefore chose to evaluate HBB in one African site and two Indian sites where our study team has ongoing prospective outcome data on all pregnancies regardless of place of delivery. In Kenya, home births account for approximately 50 % of total births, while in India most women now deliver in hospitals [8]. The primary outcome was perinatal mortality, defined in our study as the sum of all fresh, non-macerated stillbirths and early (<24 h of age) neonatal deaths within defined geographic areas, regardless of place of delivery. The rationale for this study design was 1). To test the possibility that by improving the care infants receive in facilities, an increased number of expectant mothers would choose to deliver in facilities, and 2). To demonstrate a reduction in overall neonatal mortality at the population level. If achieved, these would have a large impact on public health and hence be likely to be implemented by the health care system following the study.

The present study is the first to use a large Asian and sub-Saharan African sample, a common design, training model, resuscitation equipment, educational materials, and monitoring activities to evaluate whether HBB may have a major impact on perinatal mortality, improve implementation, and guide future policy and investment as we move toward Sustainable Development Goals [9]. The objective of this study was to assess the impact of implementing a package of HBB interventions and monitoring in select health facilities representing a large proportion of site facility births on the PMR of all registry births in Indian and Kenyan sites.

## Methods

### Study sites and population

The detailed study methods published elsewhere [8, 10–12] are summarized below. This pre-post study was conducted in three research sites of the *Eunice Kennedy Shriver* National Institute of Child Health and Human Development Global Network for Women’s and Children’s Health Research (GN). The sites included semi-urban and rural communities in Belgaum and Nagpur, India, and rural communities in western Kenya. The GN's Maternal and Newborn Health Registry (registry) was established in 2008. It is a prospective, population-based registry of all pregnancy and neonatal outcomes through 42 days postpartum in 52 clusters (defined geographic catchment areas) with 300–500 annual births. Inclusion criteria included facilities that provided delivery services 24 h per day/7 days per week and represented a significant proportion of the births in Belgaum (47 %), Nagpur (44 %), and western Kenya (38 %). Belgaum’s participating health facilities included 19 primary level (no caesarean [c]-section), 12 secondary (c-section staff on call), and 2 tertiary (c-section staff in hospital 24 h/day) facilities; Nagpur had 2 primary, 4 secondary, and 9 tertiary facilities; Kenya had 18 primary and 5 secondary facilities (Table 1).

**Table 1:** Demographic and clinical characteristics of all registry births ≥1500 g pre-post HBB intervention

	Kenya		Nagpur, India		Belgaum, India		Total	
	Pre	Post	Pre	Post	Pre	Post	Pre	Post
Registry deliveries^a^, n	8875	8394	9461	9721	16,992	16,715	35,328	34,830
Birth location, n (%)								
Hospital	1203 (13.6)	1296 (15.4)	6323 (66.8)	6995 (72.0)	12,501 (73.6)	11,931 (71.4)	20,027 (56.7)	20,222 (58.1)
Clinic	2514 (28.3)	2791 (33.2)	2966 (31.4)	2638 (27.1)	3708 (21.8)	4098 (24.5)	9188 (26.0)	9527 (27.4)
Home/Other	5158 (58.1)	4307 (51.3)	170 (1.8)	88 (0.9)	783 (4.6)	686 (4.1)	6111 (17.3)	5081 (14.6)
Birth attendant, n (%)								
Physician	209 (2.4)	203 (2.4)	5701 (60.3)	6289 (64.7)	10,446 (61.5)	10,149 (60.7)	16,356 (46.3)	16,641 (47.8)
Nurse/midwife	3617 (40.8)	3929 (46.8)	3597 (38.0)	3355 (34.5)	5838 (34.4)	5973 (35.7)	13,052 (36.9)	13,257 (38.1)
TBA	3930 (44.3)	3154 (37.6)	110 (1.2)	45 (0.5)	225 (1.3)	159 (1.0)	4265 (12.1)	3358 (9.6)
Family/unattended	1119 (12.6)	1108 (13.2)	53 (0.6)	32 (0.3)	483 (2.8)	434 (2.6)	1655 (4.7)	1574 (4.5)
Maternal education, n (%)								
No formal schooling	249 (2.8)	228 (2.7)	262 (2.8)	259 (2.7)	3091 (18.3)	2460 (14.7)	3602 (10.2)	2947 (8.5)
Primary	6174 (69.6)	5690 (68.4)	1575 (16.7)	1636 (16.9)	5300 (31.3)	5207 (31.2)	13,049 (37.0)	12,533 (36.0)
Secondary	2071 (23.3)	2019 (24.3)	5705 (60.4)	5669 (58.5)	6786 (40.1)	7005 (41.2)	14,562 (41.0)	14,693 (42.0)
University +	378 (4.3)	376 (4.5)	1899 (20.1)	2129 (22.0)	1730 (10.2)	2022 (12.1)	4007 (11.4)	4527 (13.0)
Maternal age, n (%)								
< 20	1933 (21.8)	1884 (22.7)	189 (2.0)	182 (1.9)	1650 (9.7)	1584 (9.5)	3772 (10.7)	3650 (10.5)
20–35	6595 (74.3)	6083 (73.2)	9226 (97.6)	9494 (97.8)	15,318 (90.1)	15,101 (90.3)	31,139 (88.2)	30,678 (88.3)
> 35	343 (3.9)	345 (4.1)	35 (0.37)	31 (0.32)	23 (0.14)	30 (0.18)	401 (1.1)	406 (1.2)
Parity^a^, n (%)								
0	2263 (25.5)	2232 (26.6)	4519 (47.8)	4568 (47.0)	7208 (43.5)	7253 (43.4)	13,990 (40.1)	14,053 (40.4)
1–2	3550 (40.0)	3269 (39.0)	4703 (49.7)	4892 (50.3)	8305 (50.1)	8486 (50.8)	16,558 (47.5)	16,647 (47.8)
> 2	3056 (34.5)	2883 (34.4)	236 (2.5)	258 (2.7)	1053 (6.4)	969 (5.8)	4345 (12.5)	4110 (11.8)
Labor complications, n (%)	1406 (15.8)	1269 (15.1)	1356 (14.3)	1530 (15.7)	2428 (14.3)	3050 (18.3)	5190 (14.7)	5849 (16.8)
Caesarean rate, n (%)	162 (1.8)	162 (1.9)	1879 (19.9)	2202 (22.7)	2509 (14.8)	3103 (18.6)	4550 (12.9)	5467 (15.7)
Multiple births, n (%)	99 (1.1)	102 (1.2)	57 (0.6)	68 (0.7)	122 (0.7)	103 (0.6)	278 (0.8)	273 (0.8)
Corticosteroids, n (%)								
Yes	59 (1.1)	124 (1.5)	123 (6.3)	416 (4.4)	422 (3.0)	1225 (7.3)	604 (2.8)	1765 (5.1)
No	5228 (98.9)	8209 (98.5)	1809 (92.8)	8880 (94.8)	13,746 (96.3)	15,446 (92.4)	20,783 (96.6)	32,535 (94.5)
Don’t know	0 (0.0)	3 (0.04)	18 (0.92)	73 (0.78)	103 (0.72)	44 (0.46)	121 (0.56)	120 (0.35)
Registry births, n (%)	8972	8510	9514	9784	17,109	16,815	35,595	35,109
Male, n (%)	4582 (51.1)	4281 (50.3)	4998 (52.5)	5018 (51.3)	8927 (52.2)	8687 (51.7)	18,507 (52.0)	17,986 (51.2)
Birth weight (g) ^b^, n (%)								
1500–2499	256 (2.9)	267 (3.2)	1336 (14.1)	1441 (14.7)	2261 (13.2)	3292 (19.7)	3853 (10.9)	5000 (14.3)
≥ 2500	8429 (97.1)	8145 (96.8)	8152 (85.9)	8336 (85.3)	14,824 (86.8)	13,441 (80.3)	31,405 (89.1)	29,922 (85.7)
Registry births ≥ 1500 g, n	8972	8510	9514	9784	17,109	16,815	35,595	35,109
Registry births ≥ 1500 g in facilities, n (%)	3778 (42.1)	4164 (48.9)	9342 (98.2)	9693 (99.1)	16,323 (95.4)	16,129 (95.9)	29,443 (82.7)	29,986 (85.4)
Registry births ≥ 1500 g in HBB-trained facilities, n (%)	3235 (36.1)	3481 (40.9)	4053 (42.6)	4373 (44.7)	7944 (46.4)	8131 (48.4)	15,232 (42.8)	15,985 (45.5)
Primary facility, n (%) ^c^	2620 (81.0)	2733 (78.5)	207 (5.1)	227 (5.2)	2713 (34.2)	2924 (36.0)	5540 (36.4)	5884 (36.8)
Secondary facility, n (%)	615 (19.0)	748 (21.5)	432 (10.7)	521 (11.9)	3935 (49.5)	3930 (48.3)	4982 (32.7)	5199 (32.5)
Tertiary facility, n (%)	0 (0.0)	0 (0.0)	3414 (84.2)	3625 (82.9)	1296 (16.3)	1277 (15.7)	4710 (30.9)	4902 (30.7)
Non-HBB trained facilities, n (%)	543 (6.1)	683 (8.0)	5289 (55.6)	5320 (54.4)	8379 (49.0)	7998 (47.6)	14,211 (39.9)	14,001 (40.0)

^a^ Deliveries indicate number of mothers

^b^ Birth weight measured or estimated within 7 days of delivery (*N* = 516 excluded). Birth weight values > 5500 g are excluded (*n* = 8)

^c^ Primary facilities do not perform c-sections; secondary facilities have c-section staff on call; tertiary facilities have 24 h/7 days per week c-section staff available

### Procedures and intervention

All facilities and Master Trainers received HBB training materials and equipment (Laerdal NeoNatalie® equipment and materials and clean delivery kits) based on delivery volume. The American Academy of Pediatrics HBB core staff identified best HBB training practices, assisted in developing two tiers of training workshops, and co-led the initial Master Trainer (MT) workshops. The initial single-country MT courses provided intense, hands-on training to provide at least one MT per facility in order to preserve the integrity of the intervention and expedite startup. The newly-trained MTs then conducted multiple facility-level BA team trainings with standard HBB knowledge and skills evaluation before and after the initial and refresher training courses approximately 6 months later, using the same interactive format with a maximum ratio of 6 BAs per MT. Staff turnover was addressed by providing HBB training to each new BA. The monitoring activities included direct supervision, team building and accountability measures to maintain standardized delivery room records; daily checks of equipment availability and cleanliness, daily “low dose/high frequency” [13] bag and mask ventilation practice; resuscitation debriefings and death audits; observation of deliveries or HBB skills (using a neonatal simulator if no deliveries were available) during regular and unannounced site visits; review of monthly monitoring reports and biweekly data review calls between the individual site HBB coordinators and the central core staff (RTI International and NICHD), followed by feedback to facility MTs and BAs. The pre phase was defined as the 12-month period preceding the completion of initial BA training; the post period was defined as the subsequent 12 months. Sites were also trained in basic essential newborn care [14].

### Study outcomes

The primary hypothesis was that implementation of the HBB package in facilities with substantial proportions of eligible registry newborns would decrease the PMR by 20 % among all registry births ≥1500 g in the 52 clusters. Secondary outcomes included the pre-post difference in the (1) fresh stillbirth rate (FSBR); (2) death by 1 day, including FSBs; and (3) 7-day neonatal mortality rate (NMR) among all live registry births and registry births in HBB-trained facilities. SBs were considered intrapartum or FSBs if they were not macerated (MSB). The PMR was estimated by dividing the sum of FSBs and live births dying within 7 days by all births, a modification of the common PMR definition to exclude MSBs that cannot be resuscitated. Day 1 mortality rate included FSB plus deaths by 24 h in the numerator and FSB and live births in the denominator; 7-day NMR included deaths of live births in the numerator and all live births in the denominator.

### Data management

Primary outcome and key secondary outcome data were collected by an independent registry staff. The research staff at each site collected additional data to evaluate the HBB training program and monitoring activities in the facilities.

### Power and statistical analysis

Consistent with the overall study goal outlined earlier, the study was powered to detect the overall public health impact of the intervention based on an assumption that the intervention would improve access and increase the number of facility births. We therefore estimated the power to detect the impact of implementation of HBB training in facilities to improve the mortality rates at the population (registry) level. Assuming a PMR of 25/1000 among newborns ≥1500 g, a standard deviation of 10 between clusters, and a correlation across the periods of 0.3 (based on historic registry data), the study had an estimated power of 82 % to detect a 20 % reduction in PMR among newborns ≥1500 g.

While the study was not powered to detect an interaction effect between intervention and site or to detect differences in secondary mortality outcomes, the study had sufficient sample size to provide valuable information about these secondary outcomes, e.g., the effect of the intervention based on the subset of deliveries that occurred at the HBB trained health facilities. Under the original design assumptions about heterogeneity of risk across clusters and the correlation within clusters over time, the sample sizes within the HBB-facility deliveries provided 80 % power to detect a 30 % risk reduction and a 90 % power to detect a 35 % reduction in mortality risk in these facilities. Absent significant differences in the primary outcome or this important secondary outcome, the analyses were constructed to provide point and interval estimates of the magnitude of both public health and in-facility benefit obtained from the intervention.

The primary outcome was tested using a linear mixed model that incorporated a random cluster-effect term to account for correlation within clusters across time and a fixed binary-time effect (pre-post HBB) that represented the treatment effect. The dependent variable was the cluster PMR aggregated separately across the pre and post periods. Secondary mortality outcomes were analyzed using linear mixed models, incorporating both random-cluster effects and fixed effects for pre and post periods. The interaction between site and treatment was tested for the primary and secondary outcomes. Secondary parameter estimates of combinations of time and period evaluated whether the treatment effect changed over the course of the study.

## Results

**Fig. 1:**
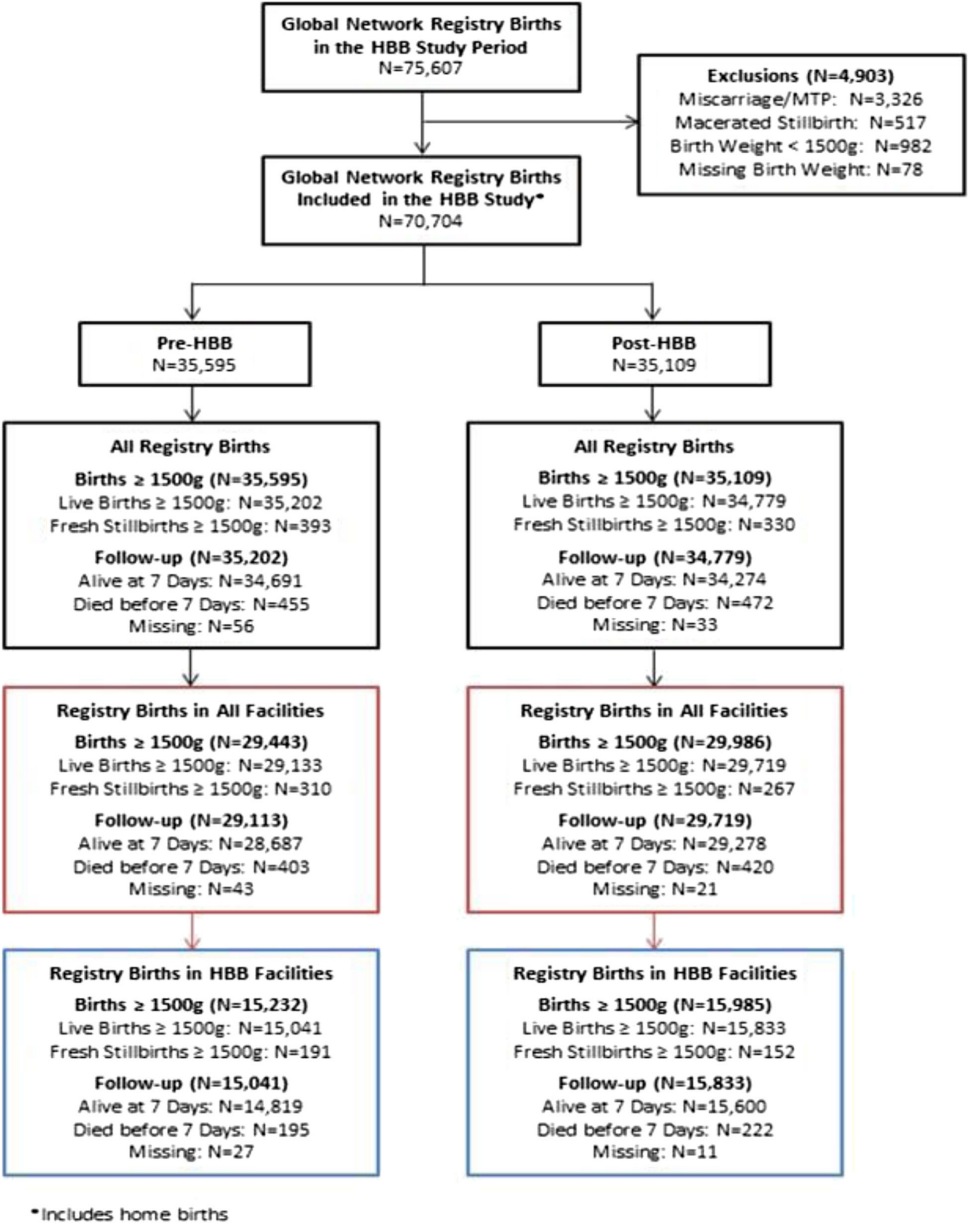
Consort diagram

The pre period was November 1, 2011 to October 31, 2012; the post period was November 1, 2012 to October 31, 2013. The 115 MTs trained 2227 BAs from June to October 2012. A total of 70,704 registry births ≥1500 g were enrolled. A majority of registry births were in facilities and 46 % of all registry births took place in the 71 intervention facilities, attended by 835 trained BAs (medical doctors, midwives, nurses, or auxiliary midwives; Belgaum = 460; Nagpur = 230; Kenya = 145). 27 % of Belgaum’s, 20 % of Nagpur’s, and 26 % of Kenya’s BAs reported receiving prior resuscitation training. Outcome data were available for 99.9 % of the births (Fig. 1).

Demographics, antenatal care, birth location, type of birth attendant, labor complications, and gender ratio were similar for the pre-post periods (Table 1); however, there was a 31 % increase in the proportion of low birthweight (LBW = 1500–2499 g) newborns in the post compared to the pre period (14.3 vs. 10.9 %) and a 22 % increase in c-sections (15.7 vs. 12.9 %), respectively.

The primary outcome was the difference in PMR among all registry births ≥1500 g pre-post HBB training (Table 2). There was no significant difference in the PMR among all registry births ≥1500 g pre-post training or in the 7-day NMR. The pre-post results for all HBB-trained facilities were similar. However, in the Kenyan HBB-trained facilities, the PMR and FSBR decreased. The pre-post changes in mortality in the HBB trained facilities in India were not significant (Table 2).

**Table 2:** Mortality among registry births ≥1500 g pre-post HBB intervention

	All Registry Births ≥ 1500 g				Registry Births ≥ 1500 g in HBB Trained Facilities			
	Pre n/N (Rate/1000)	Post n/N (Rate/1000)	Estimates^a^ of Pre-Post Differences in Mortality Rates (95 % CI)	P	Pre n/N (Rate/1000)	Post n/N (Rate/1000)	Estimates^a^ of Pre-Post Differences in Mortality Rates (95 % CI)	P
Total								
Perinatal deaths	848/35,539 (23.9)	802/35,076 (22.9)	−0.14 (−3.15, 2.86)	0.92	386/15,205 (25.4)	374/15,974 (23.4)	2.34 (−3.11, 7.80)	0.39
Fresh stillbirths	393/35,595 (11.0)	330/35,109 (9.4)	1.38 (−0.61, 3.37)	0.17	191/15,232 (12.5)	152/15,985 (9.5)	3.75 (−0.21, 7.70)	0.06
Death by day 1^b^	660/35,539 (18.6)	620/35,076 (17.7)	−0.02 (−2.51, 2.48)	0.99	295/15,205 (19.4)	297/15,974 (18.6)	0.81 (−3.65, 5.26)	0.72
Early neonatal death by day 7	455/35,146 (12.9)	472/34,746 (13.6)	−1.53 (−3.79, 0.73)	0.18	195/15,014 (13.0)	222/15,822 (14.0)	−1.41 (−5.45, 2.64)	0.49
Kenya								
Perinatal deaths	232/8947 (25.9)	202/8494 (23.8)	1.03 (−5.66, 7.71)	0.75	124/3223 (38.5)	98/3477 (28.2)	11.71 (0.39, 23.03)	0.04
Fresh stillbirths	155/8972 (17.3)	114/8510 (13.4)	2.92 (−2.21, 8.04)	0.24	83/3235 (25.7)	57/3481 (16.4)	11.27 (0.95, 21.59)	0.03
Death by day 1	204/8947 (22.8)	183/8494 (21.5)	0.25 (−5.69, 6.19)	0.93	108/3223 (33.5)	92/3477 (26.5)	8.09 (−2.05, 18.24)	0.11
Early neonatal death by day 7	77/8792 (8.8)	88/8380 (10.5)	−1.89 (−4.92, 1.14)	0.20	41/3140 (13.1)	41/3420 (12.0)	0.52 (−4.40, 5.43)	0.83
Nagpur, India								
Perinatal deaths	209/9483 (22.0)	239/9768 (24.5)	−2.92 (−8.05, 2.21)	0.25	103/4038 (25.5)	130/4367 (29.8)	−4.11 (−14.05, 5.82)	0.40
Fresh stillbirths	88/9514 (9.2)	90/9784 (9.2)	0.08 (−2.88, 3.04)	0.95	47/4053 (11.6)	57/4373 (13.0)	−1.27 (−6.80, 4.26)	0.64
Death by day 1	145/9483 (15.3)	168/9768 (17.2)	−2.11 (−5.91, 1.69)	0.26	71/4038 (17.6)	102/4367 (23.4)	−5.07 (–12.57, 2.43)	0.17
Early neonatal death by day 7	121/9395 (12.9)	149/9678 (15.4)	−3.05 (−7.53, 1.44)	0.17	56/3991 (14.0)	73/4310 (16.9)	−2.96 (−12.79, 6.88)	0.54
Belgaum, India								
Perinatal deaths	407/17,109 (23.8)	361/16,814 (21.5)	2.17 (−2.28, 6.62)	0.32	159/7944 (20.0)	146/8130 (18.0)	1.05 (−4.94, 7.04)	0.71
Fresh stillbirths	150/17,109 (8.8)	126/16,815 (7.5)	1.48 (−1.21, 4.17)	0.26	61/7944 (7.7)	38/8131 (4.7)	2.49 (−0.78, 5.76)	0.13
Death by day 1	311/17,109 (18.2)	269/16,814 (16.0)	2.33 (−1.58, 6.25)	0.22	116/7944 (14.6)	103/8130 (12.7)	0.87 (−3.88, 5.63)	0.70
Early neonatal death by day 7	257/16,959 (15.2)	235/16,688 (14.1)	0.73 (−3.50, 4.96)	0.72	98/7883 (12.4)	108/8092 (13.3)	−1.39 (−5.42, 2.64)	0.47

^a^ Estimated mean differences obtained from a mixed model with cluster as a random factor and time period as a fixed factor

^b^ Death by day 1 includes fresh stillbirths

The increase in the proportion of LBW births in the post period prompted an exploratory post hoc analysis of the mortality data stratified by birth weight (1500–2499 vs. ≥2500 g) and of the mortality data stratified by birth weight and site (Table 3). There were no significant differences in the overall registry birth outcomes pre-post training: the pre-post LBW PMR was 139.4 vs. 122.7 (*p* = 0.25); the pre-post FSBR was 72.1 vs. 55.0 (*p* = 0.16); and the pre-post 7-day NMR was 73.1 vs. 71.8 (*p* = 0.91) among all registry births. Tests of the interaction terms in the model above showed no evidence of a site by HBB intervention interaction (p > 0.33), but showed relatively strong evidence of a three-way site by HBB intervention by birth weight strata interaction (*p* < 0.0001). At the registry (population) level, the mortality rates for LBW newborns in Belgaum decreased post-intervention, with reductions ranging from 46 % in SBs to 17 % in 7-day mortality. There was no reduction in Belgaum in mortality for newborns ≥2500 g and there was no reduction for either birthweight category in Nagpur and Kenya (Table 3).

**Table 3:** Mortality among registry births ≥1500 g pre-post HBB intervention stratified by birth weight

	Birth weight 1500–2499 g				≥ 2500 g			
	Estimates^a^ of Mortality Rates (95 % CI)			P-value	Estimates^a^ of Mortality Rates (95 % CI)			P-value
	Pre	Post	Difference		Pre	Post	Difference	
All registry births	3853	5000			31,405	29,922		
Perinatal deaths	139.4 (112.9, 166.0)	122.7 (96.1, 149.2)	16.7 (−12.1, 45.5)	0.25	14.6 (12.2, 17.0)	14.3 (11.9, 16.7)	0.3 (−2.1, 2.7)	0.82
Fresh stillbirths	72.1 (51.4, 92.8)	55.0 (34.3, 75.7)	17.1 (−6.8, 41.0)	0.16	7.1 (5.4, 8.9)	6.1 (4.4, 7.9)	1.0 (−0.9, 2.9)	0.28
END^b^ by day 7	73.1 (55.4, 90.8)	71.8 (54.1, 89.5)	1.3 (−20.9, 23.5)	0.91	7.5 (6.0, 9.0)	8.2 (6.7, 9.7)	−0.7 (−2.4, 0.9)	0.38
Kenya	256	267			8429	8145		
Perinatal deaths	234.3 (174.0, 294.6)	212.5 (152.2, 272.8)	21.8 (−63.5, 107.1)	0.59	20.3 (15.0, 25.7)	18.2 (12.9, 23.6)	2.1 (−3.4, 7.6)	0.43
Fresh stillbirths	143.4 (87.4, 199.3)	107.0 (51.1, 162.9)	36.4 (−42.2, 114.9)	0.34	13.9 (9.9, 18.0)	10.6 (6.6, 14.7)	3.3 (−1.9, 8.5)	0.19
END^b^ by day 7	105.1 (58.6, 151.7)	115.3 (68.8, 161.9)	−10.2 (−76.0, 55.6)	0.75	6.5 (3.6, 9.3)	7.7 (4.8, 10.5)	−1.2 (−4.2, 1.7)	0.39
Nagpur, India	1336	1441			8152	8336		
Perinatal deaths	103.6 (78.9, 128.3)	104.6 (79.9, 129.3)	−1.0 (−24.3, 22.4)	0.93	10.5 (6.8, 14.1)	11.6 (8.0, 15.2)	−1.2 (−5.3, 3.0)	0.57
Fresh stillbirths	41.5 (25.6, 57.3)	40.6 (24.8, 56.4)	0.9 (−17.4, 19.1)	0.92	3.9 (2.2, 5.7)	4.1 (2.4, 5.9)	−0.2 (−2.7, 2.3)	0.87
END^b^ by day 7	64.2 (40.8, 87.6)	66.4 (43.1, 89.8)	−2.2 (−25.7, 21.3)	0.85	6.6 (3.8, 9.3)	7.5 (4.8, 10.3)	−1.0 (−3.6, 1.7)	0.46
Belgaum, India	2261	3292			14,824	13,441		
Perinatal deaths	89.2 (75.8, 102.6)	55.4 (42.0, 68.9)	33.8 (16.4, 51.2)	< 0.001	14.0 (11.1, 16.9)	13.8 (10.8, 16.7)	0.2 (−3.1, 3.5)	0.89
Fresh stillbirths	39.2 (30.2, 48.1)	21.1 (12.1, 30.0)	18.1 (5.5, 30.7)	<.01	4.4 (2.8, 6.0)	4.1 (2.5, 5.7)	0.2 (−1.5, 1.9)	0.78
END^b^ by day 7	52.2 (41.8, 62.7)	35.0 (24.6, 45.5)	17.2 (3.3, 31.1)	0.02	9.7 (7.1, 12.3)	9.7 (7.1, 12.3)	−0.0 (−3.6, 3.6)	1.00
HBB Facility Births	1988	2550			13,205	13,393		
Perinatal deaths	140.1 (102.9, 177.4)	102.8 (65.5, 140.0)	37.4 (−15.3, 90.1)	0.16	18.8 (14.4, 23.2)	15.9 (11.5, 20.4)	2.9 (−1.9, 7.7)	0.23
Fresh stillbirths	71.4 (40.8, 102.0)	38.1 (7.5, 68.7)	33.3 (−10.0, 76.6)	0.13	10.4 (7.0, 13.8)	7.2 (3.8, 10.7)	3.2 (−0.8, 7.2)	0.12
END^b^ by day 7	76.5 (52.2, 100.8)	66.4 (42.3, 90.4)	10.2 (−23.7, 44.0)	0.55	8.4 (5.8, 11.1)	8.8 (6.1, 11.4)	−0.3 (−3.6, 2.9)	0.84
Kenya	146	152			3073	3319		
Perinatal deaths	266.6 (160.4, 372.8)	149.2 (43.0, 255.4)	117.4 (−32.7, 267.6)	0.12	32.9 (22.4, 43.5)	22.0 (11.5, 32.6)	10.9 (−0.6, 22.4)	0.06
Fresh stillbirths	148.5 (50.1, 246.8)	48.7 (−49.7, 147.0)	99.8 (−39.2, 238.9)	0.15	24.5 (16.2, 32.8)	14.3 (6.0, 22.6)	10.2 (−1.3, 21.8)	0.08
END^b^ by day 7	143.6 (71.5, 215.8)	101.9 (32.0, 171.8)	41.7 (−58.7, 142.2)	0.39	8.6 (4.3, 12.8)	7.9 (3.6, 12.1)	0.7 (−3.0, 4.4)	0.69
Nagpur, India	628	695			3411	3676		
Perinatal deaths	100.7 (72.8, 128.6)	114.4 (86.5, 142.3)	−13.7 (−53.2, 25.8)	0.48	13.5 (7.0, 20.0)	14.2 (7.8, 20.7)	−0.7 (−8.8, 7.3)	0.85
Fresh stillbirths	44.1 (27.6, 60.7)	49.7 (33.1, 66.2)	−5.5 (−28.6, 17.5)	0.62	5.1 (1.7, 8.5)	5.6 (2.2, 8.9)	−0.5 (−5.3, 4.3)	0.83
END^b^ by day 7	59.0 (34.0, 83.9)	67.9 (42.9, 92.8)	−8.9 (−43.5, 25.7)	0.60	8.4 (2.3, 14.5)	8.7 (2.6, 14.8)	−0.3 (−8.0, 7.4)	0.94
Belgaum, India	1214	1703			6721	6398		
Perinatal deaths	62.9 (48.8, 77.1)	41.7 (27.6, 55.9)	21.2 (3.9, 38.5)	0.02	11.3 (8.0, 14.7)	12.0 (8.6, 15.3)	−0.7 (−5.0, 3.7)	0.75
Fresh stillbirths	28.5 (18.9, 38.1)	13.2 (3.6, 22.8)	15.3 (1.7, 28.9)	0.03	3.0 (1.5, 4.5)	2.3 (0.7, 3.8)	0.8 (−0.9, 2.4)	0.34
END^b^ by day 7	35.6 (26.8, 44.3)	28.9 (20.2, 37.6)	6.7 (−4.7, 18.0)	0.23	8.3 (5.5, 11.2)	9.7 (6.8, 12.6)	−1.4 (−5.5, 2.7)	0.48

^a^ Estimated means obtained from a mixed model with cluster as a random factor and time period as a fixed factor

^b^ END early neonatal death

**Fig. 2:**
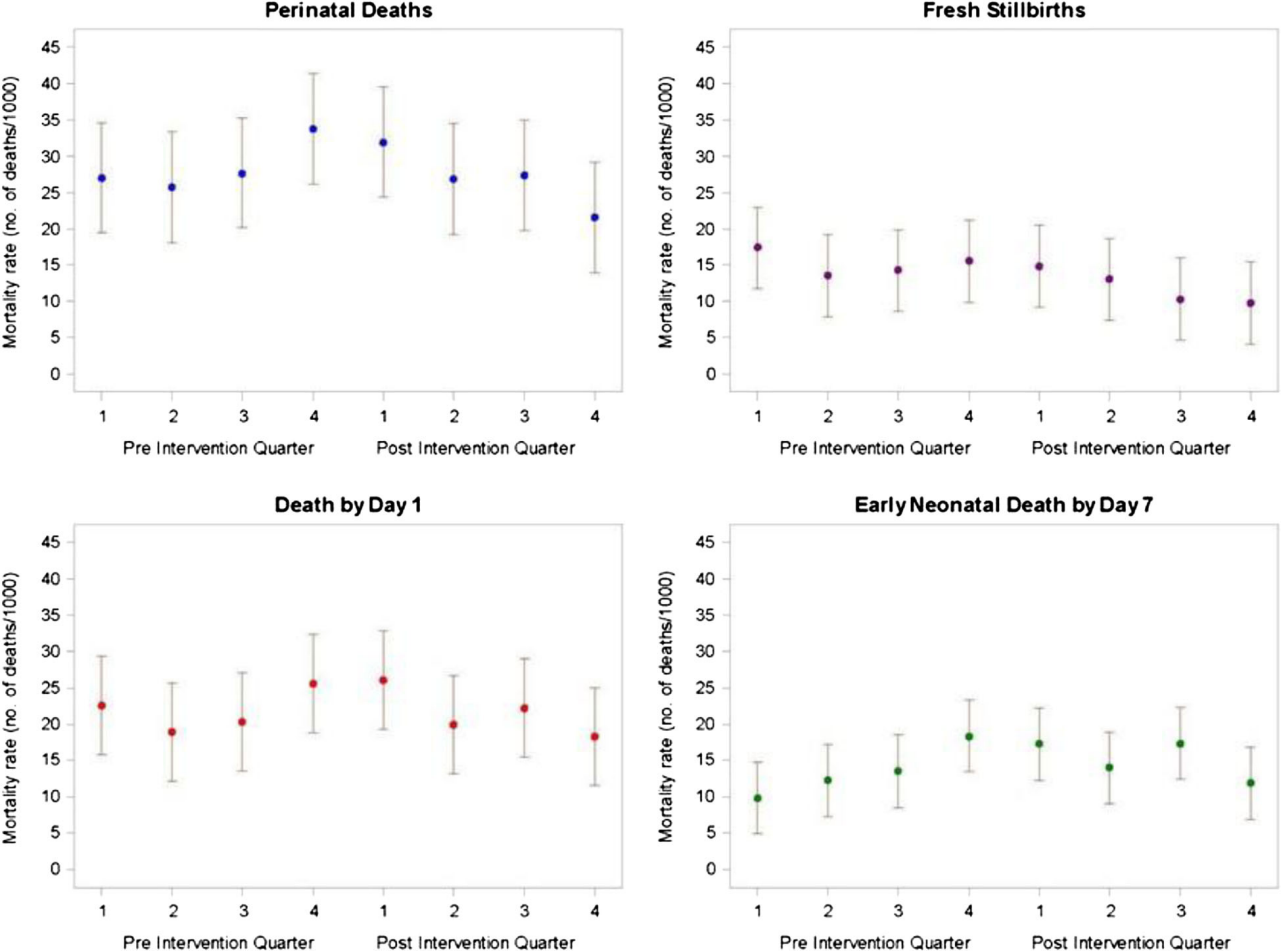
Mortality among HBB ≥1500 g registry births in HBB-trained facilities pre-post intervention by quarter

At the HBB facility level, the PMR was reduced by 34 % and the SB rate by 54 % in Belgaum in post hoc analyses, but there was no reduction in mortality rates in Nagpur. There were too few LBW newborns in HBB facilities in Kenya to provide precise estimates of changes in mortality rates. In sum, there was no reduction in mortality in normal weight newborns at any of the sites in our post hoc analyses. Finally, analysis of mortality rate over the pre-post periods by 3-month intervals in HBB-trained facilities verified that there was no change in the mortality rates beyond the expected variability of the sample (95 % CI) (Fig. 2).

## Discussion

To our knowledge, this is the first study to test a common package of resuscitation interventions, including HBB training, equipment and extensive monitoring activities, in multiple sites in sub-Saharan Africa and India. It is also the first study to identify a differential positive impact of HBB training of facility BAs on LBW neonatal survival at the population level (registry) and in HBB-trained facilities.

Our study had several strengths, including a large sample from low-income, semi-urban and rural communities in India and low-income, rural sites in sub-Saharan Africa, where most global PNM occurs. We had well-established methods for identifying and including outcomes of all delivery locations (including home births) and BA training levels. Women were enrolled starting in the second trimester; pregnancy outcomes were recorded by independent, trained staff; follow-up exceeded 99 % and the data center managed all data and data analyses. The site populations were diverse: Kenyan women, compared with Indian women, were of higher parity, and delivered heavier newborns vaginally at home with traditional BAs or in clinics assisted by nurse midwives. Finally, the HBB training was rigorous. The initial BA training was conducted by 115 MTs with facility BA teams in small, hands-on sessions with pre-post testing, followed by “refresher” training approximately 6 months later. The monitoring system was supportive and frequent. BAs that were new to the facilities were trained on an individual basis. The quality monitoring activities were also supportive and frequent, including resuscitation debriefings, death audits, daily bag-and-mask practice checks with state-of-the art equipment and training materials; scheduled and unscheduled site visits; a biweekly review of individual facility monitoring and performance data.

To evaluate the public health impact of this intervention, the primary outcome was total population mortality of neonates ≥1500g, rather than mortality of infants delivered in HBB-trained facilities, and to ensure that the public health benefit was consistent with the resource intensity of the intervention, the study was designed to detect a large effect similar to the results of the Tanzanian studies. In retrospect, the decision to test the impact of HBB training on total population mortality and to test a large effect over a relatively short time period set a high bar for the GN study, given the diversity of the sites’ health systems and the challenges of changing resuscitation behavior of all BAs within this period. Despite this high bar, the study’s design and implementation strengths provided us with an unique, unanticipated opportunity to identify a high-risk population that benefitted from the HBB intervention in both the registry (population) and HBB-trained facilities in Belgaum—LBW neonates. However, our study did not identify an overall decrease in mortality among the “normal” birthweight neonates at any of the three large sites.

Our results may not necessarily be generalizable to other countries and facility settings. Another large pre-post observational cohort study (*n* = 78,500) of HBB training in 8 Tanzanian referral hospitals included deaths among a more immature population (live births ≥750 g and FSB >1 kg) and used a brief 2-month pre (baseline) period, followed by a 1-year intensive implementation period (5 HBB MTs per facility) before assessing impact the subsequent year. The study documented a trend toward reduced deaths within 24 h and a decrease in FSB rates 1 year after the HBB implementation period, but it did not evaluate the PMR. The 2-month baseline period makes interpretation of these study results difficult; we have noted the need for longer baseline data periods because of variability in annual registry data, especially in the first two years of data collection and in times of political and facility turmoil. In addition to the duration of the baseline and implementation periods, other differences between the studies include the single vs. multi-national settings; the maturity of the birth populations; and the number and diversity of the facilities and BAs trained. Despite these differences, the GN HBB study and the Tanzanian studies shared a fundamental resuscitation strategy: both emphasized immediate drying of all non-MSBs, which focuses the BA’s full attention on immediate drying of each newborn, and uses suctioning and ventilation only “as required,” to identify FSBs.

We designed this prospective pre-post study in 71 diverse GN facilities in India and Kenya as a public health intervention to evaluate the training of BAs in selected facilities on the PMR of registry births in all the participating clusters and to improve the outcomes of all the babies born in the facilities—not just the registry babies—consistent with beliefs that facility deliveries will increase only when populations perceive that facilities provide quality care and babies survive. Although LBW survival was not identified prospectively as a secondary outcome, LBW neonates represent a high-risk population and an important indicator of quality of resuscitation and neonatal care. Stratification of data by birth weight and center identified the broad differential positive impact of HBB training on LBW survival. Mothers in both Indian sites are increasingly delivered in facilities by skilled BAs, but Nagpur’s mothers are more highly educated, are first pregnant at an older age, and have higher c-section rates than Belgaum mothers, yet HBB training did not improve the primary or secondary outcomes in Nagpur. Belgaum had relatively low baseline PMRs, FSBR, and deaths by day 1 in both their registry and HBB-trained facilities which tended to improve further after training. But what was not previously appreciated was that LBW neonates in the Belgaum community and in their HBB-trained facilities benefitted differentially from HBB training.

Based on this experience, we speculate that sites must collect a prolonged period of harmonized baseline data as the basis for demonstration of real mortality improvements. They should also provide careful monitoring of resuscitation behavior to ensure that positive behavior change is sustained, that accurate delivery room records are maintained, and that comprehensive data are analyzed promptly and made widely available to identify patterns of change. This will allow identification of high-risk populations who may be the target of increased efforts to improve quality of care. High-risk populations are especially difficult to identify in sites that are not resuscitating and recording all LBW and FSBs or are misclassifying deaths. Such problems are often identified in retrospect by an *increase* in mortality after an intervention is introduced. Time and support will be required to ensure real, sustained behavior change in highly diverse LMIC. As perinatal care improves in such settings, increased facility deliveries and ongoing resuscitation training and quality monitoring may reduce the number of MSBs, asphyxiated newborns and early neonatal deaths previously misclassified as SBs, further improving the PMR [15].

This study, like recent randomized controlled trials [16–18], highlights the complexity of scaling-up evidence-based interventions from high income countries in diverse populations and settings in LMIC. We need more information about the many subtle and striking but often unknown and unrecorded differences that make an intervention fail or succeed.

## Conclusions

Rapid scale up of HBB training of GN facility BAs was not associated with significant reductions in perinatal mortality, stillbirth, or neonatal mortality among all neonates ≥1500 g in a population-based registry in three diverse sites in India and Kenya; however, secondary analysis of outcomes stratified by BW demonstrated reductions in mortality measures in both registry and HBB-trained facilities in Belgaum with no survival benefit to normal BW populations in the three sites.

## Abbreviations

AAP, American Academy of Pediatrics; BA, birth attendant; C-section, cesarean section delivery; ENC, essential newborn care; FSB, fresh stillbirth; FSBR, fresh stillbirth rate; GN, Global Network for Women’s and Children’s Health Research; HBB, Helping Babies Breathe; LBW, newborns weighing 1500 to 2499 g; LMIC, low and middle income countries; MSB, macerated stillbirth newborns; MT, master trainer; NICHD, *The Eunice Kennedy Shriver* National Institute of Child Health and Human Development; NMR, neonatal mortality rate; PMR, perinatal mortality rate (FSB + LB dying within 7 days) ÷ all births; Registry, maternal and child health registry; SB, stillbirth
